# Rituximab - Progress but Still Not a Final Resolution for Pemphigus Patients: Clinical Report From a Single Center Study

**DOI:** 10.3389/fimmu.2022.884931

**Published:** 2022-05-03

**Authors:** Joško Miše, Ines Lakoš Jukić, Branka Marinović

**Affiliations:** ^1^ Department of Dermatology and Venereology, European Reference Network (ERN) – Skin Reference Center, University Hospital Center Zagreb, Zagreb, Croatia; ^2^ School of Medicine, University of Zagreb, Zagreb, Croatia

**Keywords:** pemphigus, rituximab, remission, relapse, efficacy, desmoglein

## Abstract

Pemphigus is a rare autoimmune disease characterized by the production of pathogenic autoantibodies against desmosomal adhesion proteins, desmoglein 1 and 3. The pathophysiological process leads to the development of blisters and erosions on mucosal and/or skin surfaces as the main clinical manifestation of the disease. Rituximab emerged as the first-line therapeutic option for pemphigus due to its ability to induce remission by depleting peripheral B lymphocytes. Our aim was to assess the efficacy of rituximab in the treatment of patients in Croatia. A single-center, retrospective study was conducted on 19 patients treated with rituximab following a rheumatoid arthritis dosing protocol between October 2015 and March 2021, with a mean follow-up of 24.1 months. After the first rituximab cycle, two patients achieved complete remission off therapy (10.5%), and six patients achieved complete remission on minimal therapy (31.6%). Partial remission was observed among ten patients (52.6%). Eight patients (44.4%) relapsed after the first rituximab cycle. The mean relapse time was 21 months. Seven patients received two rituximab cycles, and three patients received three cycles. Overall, 13 out of 19 patients experienced complete remission at some point during the study, while there were no non-responders after the rituximab treatment. No statistically significant associations were observed between age, sex, type of disease involvement and clinical remission, either on or off therapy. A steady decrease in anti-desmoglein 1 and anti-desmoglein 3 levels was measured among all patients following rituximab treatment. One patient experienced a treatment-related adverse event of infectious etiology (cellulitis). One patient died following the first rituximab cycle, with the cause of death likely not to be associated with the treatment. Rituximab is an effective disease-modifying agent in the treatment of pemphigus with the main benefit of reducing corticosteroid exposure and steroid-related side effects among pemphigus patients. However, a feature of rituximab therapy is high relapse rates and the need for repeated treatment cycles to achieve complete remission. Developing an optimal protocol for rituximab treatment and finding suitable markers for predicting relapse will improve the management of pemphigus patients.

## Introduction

Pemphigus diseases are a group of autoimmune bullous disorders characterized by the formation of autoantibodies against the desmosomal adhesion proteins, desmoglein 1 (Dsg1) and/or desmoglein 3 (Dsg3), leading to the formation of blisters and erosions of skin and mucosa. Pemphigus is a rare disease with an incidence in Croatia of 3.7 new patients per 1 million inhabitants per year (1). Before the introduction of systemic corticosteroids, the diagnosis of pemphigus was almost always a fatal one. Systemic corticosteroids and immunosuppressive drugs have drastically reduced pemphigus mortality from 75% to less than 10% in severe cases (2). The combination of prednisone/prednisolone (1.0-1.5 mg/kg/day) and corticosteroid-sparing immunosuppressive agents, mostly azathioprine and mycophenolate mofetil was regarded as a standard first-line therapy by most clinicians (3). However, severe and sometimes even life-threatening side effects related to chronic use of these drugs were still a significant issue. The increasing evidence for the successful use of rituximab was a breakthrough in the treatment of pemphigus. Rituximab, a monoclonal antibody directed against the CD20 antigen on B-lymphocytes, depletes CD20 B cells from the circulation and has been used in B-cell lymphoma, rheumatoid arthritis, vasculitides and off-label in autoimmune dermatologic conditions (4). The first case of a pemphigus patient successfully treated with rituximab was published twenty years ago (5). In a randomized controlled trial published in 2017, Joly et al. showed that 89% of patients with pemphigus vulgaris (PV) and pemphigus foliaceus (PF) assigned to the rituximab group achieved complete remission off therapy compared to 34% of patients assigned to the treatment with prednisone alone (6). In 2018 rituximab was licensed for the treatment of moderate to severe pemphigus in the United States and the European Union. More recently, the consensus statement on management of pemphigus by the international panel of experts and the guidelines by the European Academy of Dermatology and Venereology (EADV) recommended intravenous CD20 inhibitors as a first-line therapy option for mild and moderate-to-severe pemphigus (7, 8). In the years following, a number of studies evaluating the efficacy and outcomes of rituximab therapy for pemphigus were published. This study aimed to assess the efficacy of rituximab in treating patients with pemphigus in Croatia and compare our results with the published studies on rituximab effectiveness from other centers.

## Materials and Methods

### Patients

We identified eligible patients from treatment logs at the University Hospital Center Zagreb, the Croatian Referral Center for Bullous Dermatoses. The study included all patients with PV and PF treated with rituximab from October 2015 to March 2021. Nineteen patients were identified, out of which 16 were diagnosed with PV and 3 with PF. Patient characteristics are summarized in **Table 1**. A diagnosis of PV and PF was based on the clinical appearance of mucosal and/or cutaneous lesions and confirmed by the histopathological finding of suprabasal (PV) or subcorneal (PF) acantholysis and direct immunofluorescence results of intercellular immunoglobulin G, with or without C3 deposits, in the epidermis/epithelium.

**Table 1:** Baseline characteristics of patients.

Patients included (No.)	19
M/F (No.)	5/14
Mean age (Years)	55.3 (M: 55.2, F: 55.4)
Previous therapies (No.)	Prednisone (19) Azathioprine (16)
RTX dosing protocol (No.,%)	Rheumatological (19, 100%)
Anti-Dsg 1 mean, baseline (U/mL)	123.45
Anti-Dsg 3 mean, baseline (U/mL)	197.30
Time interval between diagnosis and rituximab administration (Months)	Mean 80.57, range 9-221
Follow-up (Months)	Mean 24.1, range 6-65

### Treatment

Patients received an initial intravenous (IV) infusion of 1000 mg rituximab on day 1 and a second IV infusion of 1000 mg rituximab on day 15 (rheumatoid arthritis protocol). Each patient received rituximab while being hospitalized at our Department. Before starting rituximab treatment, all patients underwent general, and laboratory examination and vaccinations were administered as indicated (8). Patients received premedication 30 minutes prior to rituximab infusion with paracetamol 1g IV, loratadine 10 mg *per os* and methylprednisolone 125 mg IV. Vital parameters of each patient were monitored during and after rituximab infusion. Patients who relapsed were treated with an additional cycle of 2g of rituximab combined with reintroduced or escalated prednisone dose. First-line treatment in all patients included oral prednisone at an initial dosage of up to 1 mg/kg/day tapered off over 6-12 months. In 16 out of 19 patients, a steroid-sparing drug (azathioprine) was also introduced at the dosage between 0.5 mg/kg/day and 2.5 mg/kg/day depending on the activity level of the thiopurine methyltransferase (TPMT) enzyme.

### Clinical Response and Adverse Events Assessment

Clinical response was defined by the criteria outlined in the consensus statement on definitions of endpoints and therapeutic response for pemphigus (9). Complete remission off therapy (CROT) was defined as complete epithelialization and absence of new or established lesions while the patient is off all systemic therapy for at least 2 months while complete remission on minimal therapy (CRMT) was defined as the absence of new lesions while the patient is receiving minimal doses of systemic therapy. Partial remission off therapy (PROT) was defined as the presence of transient new lesions that healed within 1 week without treatment and while off all systemic therapy. Minimal therapy was defined as prednisone up to 10 mg/day or azathioprine up to 1.25 mg/kg/day. Relapse was defined as the appearance of at least three new lesions in 1 month that did not heal spontaneously within 1 week or the extension of established lesions in a patient who had previously achieved disease control. Anti-desmoglein 1 (anti-Dsg 1) and anti-desmoglein 3 (anti-Dsg 3) antibody titers were measured at the time of pemphigus diagnosis, before the start of rituximab treatment (baseline) and at months 3, 6 and 12 after receiving rituximab. The primary study outcome was CROT six months after one rituximab cycle. Secondary study outcomes included CROT six months after additional rituximab cycles, levels of anti-Dsg 1 and anti-Dsg 3 titers after one rituximab cycle, relapse rates, the median time to relapse and incidence of treatment-related serious adverse events. Serious adverse events were defined according to the Food and Drug Administration (FDA) definition as any event that is fatal or life-threatening, requires hospitalization or causes disability or permanent damage.

### Study Design

We conducted a single-center retrospective study of 19 patients with PV and PF treated with rituximab in our center from October 2015 and March 2021 with at least 6 months of follow-up. This study was conducted in accordance with the Declaration of Helsinki and was approved by the Committee of Ethics of the University Hospital Center Zagreb. The requirement for the acquisition of informed consent was waived due to the study’s retrospective design.

### Statistical Analysis

Data were analyzed using statistical packages STATISTICA ver. 12 (StatSoft, Inc., Tulsa, OK, USA) and MedCalc^®^ Statistical Software ver. 20.015 (MedCalc Software Ltd, Ostend, Belgium). Categorical variables were presented as numbers and proportions (%) and continuous variables as mean with standard deviation (SD) or as median with interquartile range (IQR) depending on the distribution. Categorical data were tested using a chi-square test for the differences between groups. Repeated measures ANOVA was used to test for differences in time dynamics of outcome measures between groups. Kaplan-Meier survival analysis was used to test for differences in time to the first event between groups. Logistic regression analysis was used to assess associations with different outcomes. ROC analysis was used to assess the pretreatment level of anti-Dsg with outcomes. P<0.05 was considered statistically significant for all tests, corrected for multiple comparisons.

## Results

A total of 19 patients, 16 of them with PV and 3 with PF, received the first cycle of rituximab. There were 14 female and 5 male patients. The median age was 55.3 (M: 55.2,F: 55.4) and the mean disease duration before administration of rituximab was 80.57 months with range from 9 to 221 (**Table 1**). Two patients achieved CROT (10.5%), six patients achieved CRMT (31.6%), and PR was observed in 10 out of 19 patients (52.6%). One patient died during the follow-up after the first rituximab cycle. Overall, significant improvement was observed in 8 of 19 patients (42.1%).

**Figure 1:**
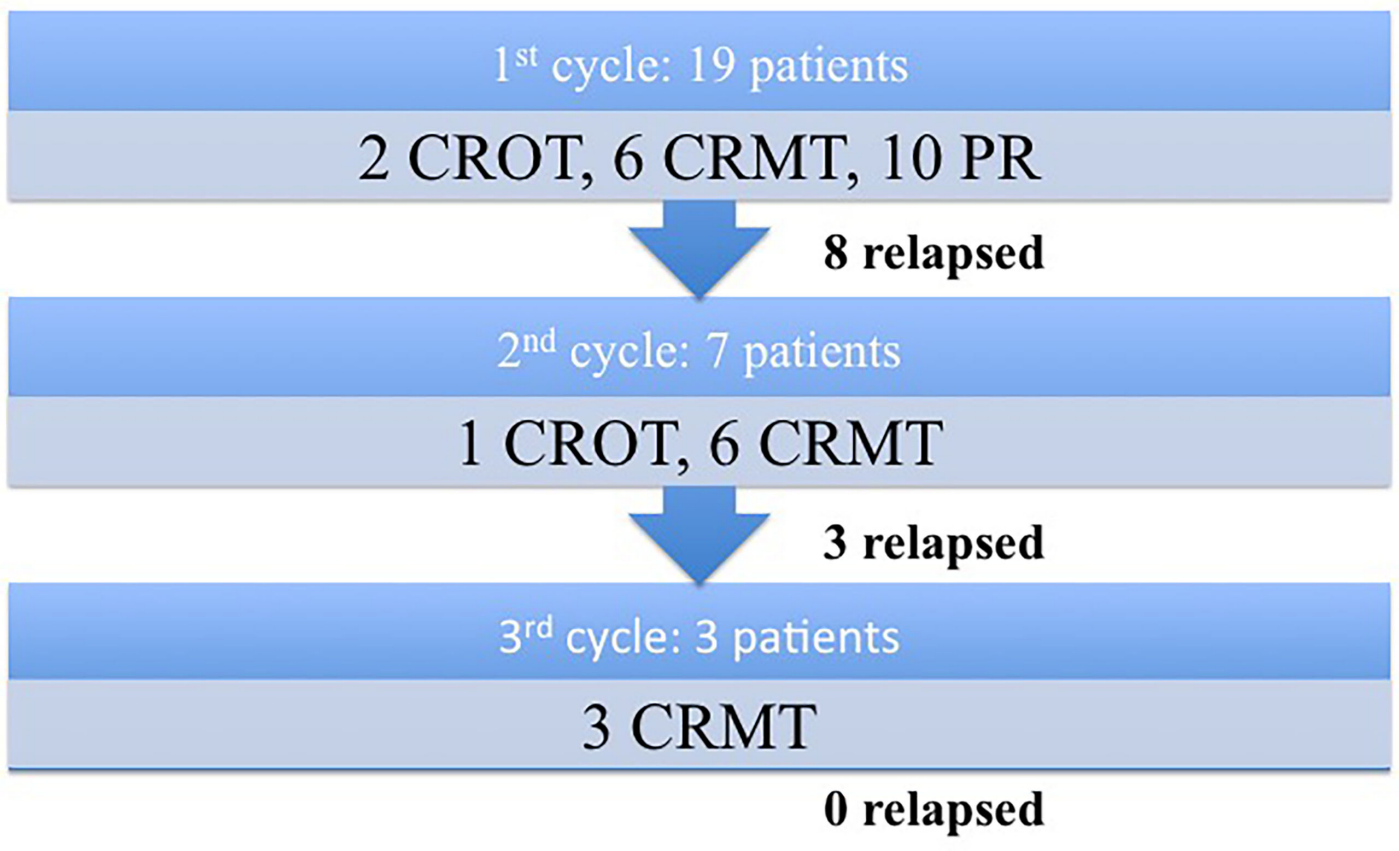
Flowchart describing patients’ clinical response following the administration of rituximab treatments.

Eight patients (44.4%) relapsed after the first cycle (p=0.0395); 4 patients who were in PR and 4 patients who were in CR. The mean relapse time was 21 months (range: 6-50). Seven of these eight patients went through the second cycle of rituximab. Six achieved CRMT (85.7%), with one patient achieving CROT (14.3%). Among these seven patients, three relapses were observed after the second cycle (42.8%). They were retreated with a third cycle of rituximab, and all of them achieved CRMT. There were no relapses observed among patients who received the third rituximab cycle by the end of the inclusion period of this study. **Figure 1** represents a flowchart describing patients’ clinical response following the administration of rituximab treatments.

The mean remission length after the first rituximab cycle was 20 months. Overall, 13 out of 19 patients experienced complete remission at some point during the study, while there were no non-responders after the rituximab treatment. Patients achieving complete remission had a mean disease duration prior to rituximab of 67 months, against 82 months in those not achieving complete remission. However, the difference was not statistically significant (p=0.40). There were no other statistically significant associations between age, sex, type of disease and clinical remission, either on or off therapy. Patient demographics, including sex and type of disease were not significant predictors of relapse.

All patients in every rituximab cycle were concomitantly receiving corticosteroids with the dose gradually tapered depending on the clinical status. The mean corticosteroid dose per day decreased progressively with each new cycle (31 mg in the first cycle, 21 mg in the second cycle, and 16 mg in the third cycle). Sixteen patients received adjuvant immunosuppressive (azathioprine) during the first cycle. Four patients continued the medication throughout the second, and only one patient received the adjuvant in the third cycle with achieved complete remission on minimal therapy (**Table 2**).

**Table 2:** Concomitant therapy during each rituximab cycle with patient outcomes.

Cycle	No. patients	No. patients receiving concomitant steroid dose	Mean concomitant steroid dose per day	No. patients receiving adjuvant*	No. patients in CROT	No. patients in CRMT	Relapsed
1^st^	19	19/19 (100%)	31 mg	16/19 (84.2%)	2/19 (10.5%)	6/19 (31.5%)	44.4%
2^nd^	7	7/7 (100%)	21 mg	4/7 (57.1%)	1/7 (14.3%)	6/7 (85.7%)	42.8%
3^rd^	3	3/3 (100%)	16 mg	1/3 (33.3%)	0/3	3/3 (100%)	0%

*Azathioprine 100 mg/day.

**Figure 2:**
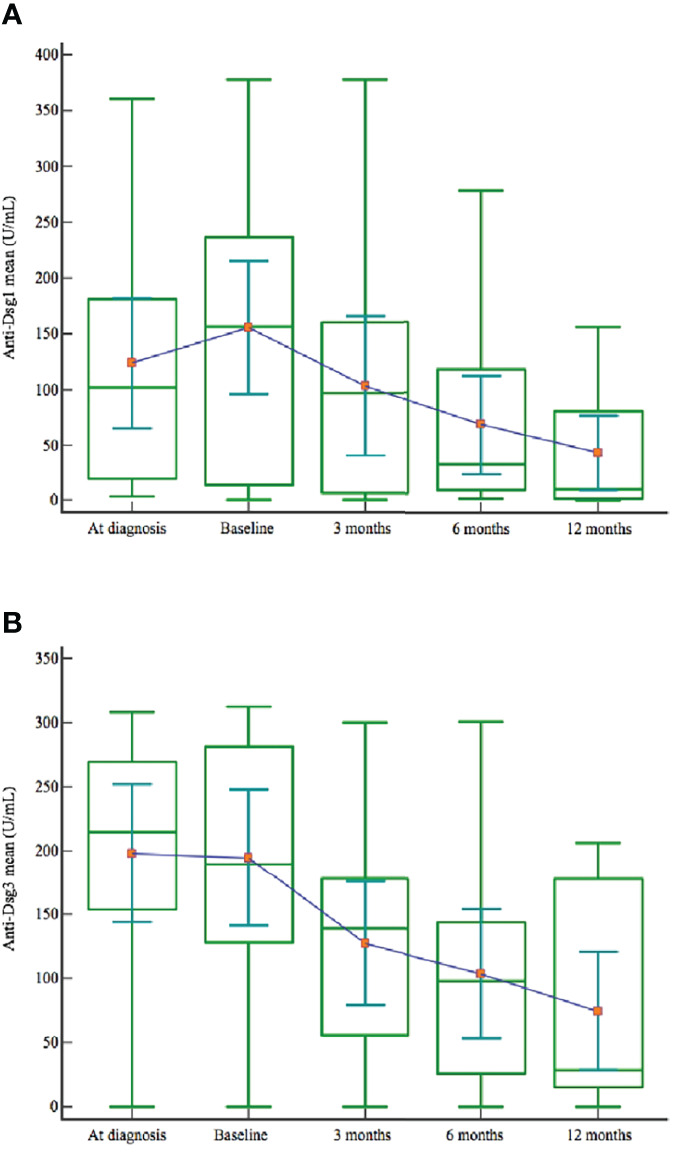
Anti-Dsg 1 **(A)** and anti-Dsg 3 **(B)** mean values at the diagnosis, before the start of retuximab and at months 3, 6 and 12 after rituximab treatment.

We recorded a steady decrease in anti-Dsg 1 and anti-Dsg 3 levels after rituximab treatment among all patients (**Table 3**). Mean anti-Dsg 1 and anti-Dsg 3 values dropped at months 3, 6 and 12 following rituximab infusion (**Figure 2**).

**Table 3:** Descriptive statistics for anti-Dsg 1 and anti-Dsg 3.

	Minimum	Maximum	Median	95% CI	25 - 75 P
Anti-Dsg1 bf RTX	0.000	377.780	156.465	46.447 to 235.652	13.410 to 236.49
Anti-Dsg1 3 months after	0.000	377.780	96.540	6.116 to 160.407	6.057 to 160.44
Anti-Dsg1 6 months after	1.560	277.620	32.530	9.215 to 116.936	9.203 to 117.32
Anti-Dsg1 12 months after	0.000	155.300	10.460	1.561 to 78.970	1.558 to 79.960
Anti-Dsg3 at DG	0.000	390.270	214.295	132.719 to 268.772	118.930 to 269.40
Anti-Dsg3 bf RTX	0.000	312.240	205.710	129.237 to 265.071	127.930 to 269.12
Anti-Dsg3 3 months after	0.000	300.030	121.610	36.907 to 175.690	36.533 to 175.82
Anti-Dsg3 6 months after	0.000	300.030	97.180	22.577 to 137.768	22.510 to 138.09
Anti-Dsg3 12 months after	0.000	205.340	28.275	14.779 to 178.349	15.490 to 178.00

There were no statistically significant associations observed between the baseline levels of anti-Dsg 1 or 3 antibodies and CROT, CRMT or both (p=0.44, p=0.28, p=0.64). No statistically significant association was observed between anti-Dsg 1 or 3 levels and relapse after rituximab treatment (p=0.38).

One adverse event (5.2%) was observed that we attribute to rituximab treatment. A female patient developed cellulitis that was treated with systemic antibiotics. One patient died in the first year of completing a rituximab cycle. However, the cause of death (acute myocardial infarction) is most likely not attributable to rituximab treatment. We did not record any adverse events related to the infusion of rituximab. No serious adverse events, as defined by the FDA, were observed. However, there could be more adverse events that we failed to capture because of the retrospective design and less reporting of side effects as many get treated in the primary or secondary care units. Furthermore, it is difficult to attribute adverse events to rituximab, given that patients were concomitantly receiving other immunosuppressive therapies.

## Discussion

Our study showed that rituximab is effective in inducing remission as 68% of pemphigus patients who received rituximab achieved a complete remission at some point during the follow-up period. However, more than one cycle of rituximab therapy is needed to achieve the desired treatment outcome. After the first cycle, complete remission, off or on minimal therapy, was achieved in 31.5% of patients, and after the second rituximab cycle, all patients achieved complete remission, either off or on minimal therapy. This finding is consistent with almost all studies involving the assessment of rituximab efficacy, showing that it effectively induces remission, but with more than one administered cycle, ranging from two to as many as seven in some patients (10).

Rituximab has, until 2020, largely been used as a second or third-line treatment option for PV, and we have used it as such at our Department for the patients involved in this study. However, it has recently been recommended by the international panel of experts, as well as by European guidelines, as a first-line treatment for PV. Among a number of studies related to the use of rituximab as the first *vs* second-line treatment, several studies reported a higher probability of achieving complete remission when rituximab is used as a first-line agent (11–14). However, contesting these findings are studies that observed no statistically significant difference in achieving complete remission between the two groups (15–18). Among them is a systematic review by Amber et al. reporting no association between the number of previous treatments and clinical outcomes (19). It needs to be highlighted that the findings of the superior effect of rituximab when administered as the first-line therapy may be influenced by the possible presence of more recalcitrant patients who previously relapsed and therefore received rituximab as the second or third-line agent. The absence of Pemphigus Disease Area Index (PDAI) score in the reviewed studies makes it difficult to assess disease severity among patient cohorts who received rituximab as first or second-line treatment impeding us to provide a definite answer to this question. However, studies are showing that patients who received rituximab earlier during the course of their disease had a higher chance of achieving complete remission. Lunardon et al. reported that patients in complete remission had a median disease duration of 19 months compared to 86 months in those not achieving complete remission (20). Furthermore, Balighi et al. found better outcomes in patients treated with rituximab within 6 months of diagnosis (12). In our study, patients achieving complete remission had a mean disease duration of 67 months prior to rituximab therapy, against 82 months in those not achieving complete remission. However, the difference was not statistically significant. The long-term follow up in most published reports cover a period of only about three years after the first rituximab cycle. The study by Shimanovich et al., published in 2020, followed the patients for a notably longer time, with a median of 104 months (8 years and 8 months). They reported that 95% of patients achieved a complete remission at some point, with about 27% of patients achieving long term complete remission off therapy and relapses observed in two-thirds of the patients, as late as 156 months after a successful rituximab cycle (15).

In our study 8 patients (44.4%) relapsed after the first rituximab cycle, which is consistent with the findings from other studies. Wang et al., in their meta-analysis from 2015, reported relapse rates of 2%, 14% and 40% at 6-month, 12-month and overall (21). Conducting the literature review, we found that the relapse rate after the first rituximab cycle ranged from 24% to 65%. Curimbhoy et al. reported that 65% of patients relapsed after the first rituximab cycle, 18% relapsed after the second cycle and 20% after the third administered cycle (22). A similar “crescendo effect” was observed in the study by Shimanovich et al. with a 63% relapse rate after the first rituximab cycle and 41% and 43% after the second and third cycle, respectively (15). We found that 44.4% of our patients relapsed after the first rituximab cycle, 42% after the second, and none after the third cycle supporting the observation that repeated rituximab cycles lead to progressively decreasing relapse rates. The overall relapse rate is difficult to narrow down because of the differences in the follow-up time between the studies. The mean relapse time following rituximab treatment among our patients was 21 months, with one patient relapsing at month 50 after a rituximab cycle. In the literature, the mean relapse time following rituximab therapy was reported between 8 and 24 months (21).

Recent research has also focused on finding relevant prognostic factors for predicting clinical remission and relapse. Assessing the correlation between age, sex, anti-Dsg titer levels, disease duration, and the outcomes of rituximab therapy is of practical interest. With the collected data from our study, we analyzed anti-Dsg 1 and 3 values and tried to see whether the titer levels could serve as an indicator for predicting the clinical outcome. Even though anti-Dsg 1 and 3 levels progressively decreased following each rituximab cycle, we could not establish a statistically significant association between anti-desmoglein titer levels and remission or relapse. The existing data from different studies is somewhat conflicting regarding the role of anti-Dsg 1 and anti-Dsg 3 in predicting treatment outcomes and relapse. In recent years, some studies suggested anti-Dsg 1 as a more valuable marker of clinical outcome than anti-Dsg 3 in pemphigus patients (12, 23). However, the consensus is lacking, as there are studies that find no statistically significant difference between anti-Dsg 1 and 3 in predicting a favorable clinical outcome and relapse (11). The study by Albers et al., which focused on identifying biomarkers predictive of relapse, found that anti-Dsg 3 level had a strong predictive value for relapse among all patients and that positive anti-Dsg 1 level had significant predictive value among patients with the mucocutaneous disease, which is contrary to the previous findings of the role of anti-Dsg1 in pemphigus phenotype (24). This should come as no surprise as there is increasing evidence that antibody specificities and titers do not always correlate to the disease activity and clinical features of pemphigus. Some studies suggest that these discrepancies account for between 36% and 48% of all pemphigus cases (25). The reason for these cases that challenge “desmoglein compensation theory” could be in a distinct set of antibodies to desmoglein and various non-desmoglein antigens (desmocollin, plakins, armadillo proteins, cholinergic receptors, hSPCA1 and antimitochondrial proteins) that each patient develops during the course of the disease (26, 27). Antibodies against these non-desmoglein antigens could maintain disease activity, casting doubts at the attempts to use anti-Dsg 1 and 3 titer levels to predict relapse and remission successfully. Kushner et al., when controlling for age and dosing protocol, revealed that older age (65 and older) was significantly associated with achieving complete remission after rituximab therapy (10). The explanation given for this finding could be in the weakened immune system in elderly patients, making remissions of autoimmune diseases easier to achieve. Toosi et al. analyzed the differences between PDAI scores in patients with or without relapse and found that patients with higher PDAI scores, especially higher mucosal PDAI scores at baseline, may have a higher risk of relapse in the future (11).

It is evident that relapse poses a problem after treatment with rituximab. A plausible explanation for high relapse rates could be in the existence of ectopic lymphoid structures within pemphigus lesions that consist of T and B lymphocytes in various stages of differentiation. The question remains if anti-CD20 treatment substantially depletes B-cells in the pemphigus lesions as the depth of B cell depletion depends on the target tissue (28). Recent findings suggest that locally present ectopic lymphoid structures evade the systemic depletion induced by rituximab and facilitate the resistance of lesions even in the absence of circulating Dsg-autoantibodies. Furthermore, the study by Zhou et al. detected a much higher fraction of Dsg-specific B cells in pemphigus lesions than in peripheral blood, indicating that pemphigus lesions could be a reservoir of B-cells maintaining the disease activity (29). These findings could provide an explanation for relapses after rituximab treatment and a possible future therapeutic target - chemokines that facilitate the migration of B lymphocytes into the skin, or a different treatment modality - intralesional rituximab. Furthermore, hematological dosing protocol for rituximab administration shows a deeper B-cell depletion in the secondary lymphoid tissues than the rheumatoid arthritis dosing protocol (30). Several studies confirm this finding by reporting lower relapse rates among patients receiving hematological dosing protocol (375 mg/m2 body surface area, four infusions one week apart) (22, 31, 32). However, concerns remain regarding the safety and consequences of a complete B-cell clone eradication (19).

Identifying the patients who are more likely to relapse or have a poor response to therapy is beneficial for determining the optimal dosage and timing of maintenance therapy. Eight of our patients who relapsed after the first cycle relapsed between month 6 and month 50 (median: 16 months), which gives clues on the best timing of maintenance rituximab infusion. Considering that half of our patients relapsed in the second year of follow-up, it seems reasonable to give patients a maintenance dose of rituximab at month 12 to prevent these relapses. However, results are lacking on the treatment outcomes of patients who received maintenance therapy making valid counterfactual reasoning if patients who received maintenance therapy would have achieved the same relapse rates. Nevertheless, EADV guidelines on pemphigus management from 2020 recommend maintenance infusion of rituximab at month 6 for patients with severe pemphigus and/or who still have high titers of anti-Dsg antibodies, whereas maintenance infusion at month 12 is considered for all patients in complete remission and in particular for those who have positive anti-Dsg antibodies (7).

A challenge in assessing the treatment outcomes of rituximab is the heterogeneity of definitions, follow-up duration and patient data available among different studies. In order to streamline the reports on rituximab efficacy which would allow for a more robust and reliable comparison between various studies, we recommend following the consensus statement on definitions of disease and endpoints (9). Collecting patient data, such as PDAI scores, and measuring anti-Dsg 1 and 3 levels at 3-month intervals, could provide valuable information in predicting and preventing a relapse, which still comprises a significant problem in the management of pemphigus patients. Further research is needed to establish the optimal rituximab dosing protocol taking into account clinical outcomes, safety and cost-effectiveness of anti-CD20 therapy.

Limitations of our study are the absence of PDAI and/or ABSIS scores and small sample size. Due to the study’s retrospective nature, PDAI or ABSIS scores were not available for all patients, so we decided not to include them in the study. The number of patients is small, but in accordance with the number of newly diagnosed pemphigus patients in Croatia and the number of patients with severe pemphigus referred to our center. Despite these limitations, our study provides important information on the long-term effects of rituximab in a well-defined group of patients treated in a single center with the same treatment regimens.

## Conclusion

The results of our study show that rituximab is generally well tolerated and effective in inducing remission among pemphigus patients. The indispensability of rituximab lies in its ability to significantly decrease corticosteroid exposure and corticosteroid-related side effects, making it major progress for pemphigus patients. However, a feature of rituximab therapy is high relapse rates and the need for repeated treatment cycles. The presence of ectopic lymphoid structures in pemphigus lesions and the variety of antibodies involved in the pathophysiology of pemphigus make it difficult to establish a one-size-fits-all approach in the management of pemphigus. Furthering the research into understanding the complete autoantibody profile, not only antidesmosomal, will help establish a personalized pemphigus subtype for each patient, which will open new therapeutic opportunities.

## Data Availability Statement

The raw data supporting the conclusions of this article will be made available by the authors, without undue reservation.

## Ethics Statement

The studies involving human participants were reviewed and approved by Committee of Ethics of the Medical School University of Zagreb. Written informed consent for participation was not required for this study in accordance with the national legislation and the institutional requirements.

## Author Contributions

JM, IJ, and BM contributed actively to the preparation of the manuscript. Conceptualization: JM; Writing - original draft preparation: JM, IJ, and BM; Writing—review and editing: JM, IJ, and BM; Figures: JM; Collecting sources of information: JM, and IJ; Supervision: BM and IJ. “All authors contributed to the article and approved the submitted version.

## Conflict of Interest

The authors declare that the research was conducted in the absence of any commercial or financial relationships that could be construed as a potential conflict of interest.

## Publisher’s Note

All claims expressed in this article are solely those of the authors and do not necessarily represent those of their affiliated organizations, or those of the publisher, the editors and the reviewers. Any product that may be evaluated in this article, or claim that may be made by its manufacturer, is not guaranteed or endorsed by the publisher.
